# Impact of age and gender differences in the prevalence and patterns of multimorbidity in the Thai Cohort Study

**DOI:** 10.1093/inthealth/ihae018

**Published:** 2024-03-23

**Authors:** Xiyu Feng, Haribondhu Sarma, Sam-ang Seubsman, Adrian Sleigh, Matthew Kelly

**Affiliations:** Department of Applied Epidemiology, National Centre of Epidemiology and Population Health, the Australian National University, Canberra 2601, Australia; Department of Applied Epidemiology, National Centre of Epidemiology and Population Health, the Australian National University, Canberra 2601, Australia; School of Human Ecology, Sukhothai Thammathirat Open University, Nonthaburi, 11120, Thailand; Department of Applied Epidemiology, National Centre of Epidemiology and Population Health, the Australian National University, Canberra 2601, Australia; Department of Applied Epidemiology, National Centre of Epidemiology and Population Health, the Australian National University, Canberra 2601, Australia

**Keywords:** Age, chronic conditions, gender, multimorbidity

## Abstract

**Background:**

The study aims to identify the common patterns of multimorbidity and their distribution by age and gender.

**Method:**

This cross-sectional study collected self-reported data from 42 785 Thai Cohort Study members through mailed questionnaires. Employing prevalence-based analysis, it identified common multimorbidity (coexistence of two or more chronic conditions) patterns, analysing the three most common patterns stratified by age and sex. P for trend (p-trend) was used to test the linear trend for associations between age and prevalence of these chronic conditions in the multimorbidity patterns.

**Results:**

Chronic conditions with the highest prevalence were related to metabolic syndromes: obesity (28.5%), hyperlipidaemia (13.2%) and hypertension (7.2%). A positive linear age–multimorbidity association was observed (p-trend = 0.0111). The 60+ participants averaged 1.20 diseases, with 33.7% multimorbidity prevalence. Hyperlipidaemia + obesity was most prevalent in the under-40 multimorbid group (38.7%). Men exhibited a higher prevalence of multimorbidity and associated patterns involving hypertension, hyperlipidaemia and obesity than women.

**Conclusion:**

Metabolic syndrome components were the prominent factors driving multimorbidity. Significant age and gender differences were also revealed in multimorbidity prevalence. People aged 60+ faced high risk of multimorbidity, while younger individuals tended towards the multimorbidity pattern of obesity and hyperlipidaemia. Men were more susceptible to multimorbidity patterns associated with metabolic syndromes. Future studies for metabolic-related multimorbidity should consider these differences, addressing age and gender issues.

## Introduction

Multimorbidity, where the same person has two or more chronic health problems at the same time, is a growing global burden, and ageing is the most important risk factor.^1,2^ In Southeast Asia, the burden of non-communicable diseases (NCDs) has risen as economic levels have improved and life expectancy has increased. Ageing populations are also more likely to experience multimorbidity, which has led to a gradual increase in global prevalence from roughly 4.5% at the beginning of the twenty-first century to 10% currently.^3^ A study from Indonesia showed a prevalence of about 20.8% in 2014.^4^

Effective prevention and treatment can only be carried out if the patterns of multimorbidity are identified. Currently, common multimorbidity patterns include cardiometabolic multimorbidity, mental-physical multimorbidity, metabolic conditions and cancer, which may be related to ageing and changes in lifestyle.^1,5,6^ However, in the countries of Southeast Asia, studies of these patterns of multimorbidity are relatively scarce.^1–3^

Like other countries in Southeast Asia, after more than half a century of change from agrarian to industrialization, the population in Thailand is undergoing a health transition. NCDs have replaced infectious diseases as the number one killer of Thai people.^7^ Chronic conditions associated with metabolic syndromes, including obesity, hyperlipidaemia and hypertension, are becoming increasingly common, as are NCDs including cardiovascular diseases (CVDs) and diabetes. For example, from 2005 to 2019, disability-adjusted life year (DALY) loss due to CVDs increased by about 480 000, and DALY loss due to diabetes increased by about 370 000.^8^ However, there is a lack of research on multimorbidity in Thailand, with most studies focusing on single or specific diseases, while the presence of unrelated or uncoordinated multiple chronic diseases has not been studied in detail.^3–6^

The purpose of this paper is to improve understanding of the prevalence of multimorbidity and the common patterns of multimorbidity in different ages and genders in Thailand. It may help health-care decision-makers and researchers in better understanding the co-occurrence of diseases to design appropriate and targeted prevention and treatment to deal with multimorbidity.

## Materials and Methods

### The study sample

#### The Thai Cohort Study

The Thai Cohort Study (TCS), was established in 2005, studying 87 134 Thais (aged 15–87 years old) enrolled at Sukhothai Thammathirat Open University (STOU) who lived across the country. The only participant criterion was to be registered students of STOU.^9–11^ Sampling method, sample size calculation and design effect are not applicable to the TCS. The TCS was a study where the questionnaires were distributed to a population of around 200 000 students from STOU. The objective was to include as many participants as possible to ensure comprehensive coverage.^9^ The students who responded (around 45%) formed the baseline cohort study group. The main aim of this study was to identify relationships between exposures and outcomes in the cohort (internal validity) rather than claiming that the results represent prevalence levels in the general Thai population (external validity). Our study is based on this 2013 endpoint of the TCS.^9^

Our study primarily relies on the data collection methods and overall research design of the TCS.^9^ This study is a cross-sectional analysis of 42 785 respondents who completed the final questionnaire in the TCS in 2013. As well as information on diagnosis with chronic conditions, the extracted data included age (divided into three groups: up to 39 y, 40–59 y, and ≥60 y) and gender (male and female).

### Multimorbidity prevalence and patterns

In the 2013 mail-in questionnaire, one of the questions was whether respondents had been diagnosed by a doctor with any of the 11 chronic diseases listed, with those who had these diseases reporting ‘yes’ and those without them reporting ‘no’.^9^ Furthermore, participants also self-reported their weight and height, and body mass index (BMI) was calculated from these two variables. Obesity was then defined as having a BMI greater than 25 kg/m^2^, which was classified as ‘yes’, and a BMI less than 25 kg/m^2^ classified as ‘no’.^9,10^ A BMI over 25 kg/m^2^ was defined as obese according to the specific cut-off for Asian populations, rather than the general WHO definition of over 30 kg/m^2^ as obese.^10^

There were 12 conditions in this study (see Table 2). Multimorbidity was defined as the same individual having two or more of these 12 chronic conditions being reported as ‘yes’. The chronic conditions included cardiometabolic conditionxs (hyperlipidaemia, hypertension, ischaemic heart disease and stroke), cancers (of liver, lung, stomach, colon-rectum and breast) and other conditions (kidney diseases and obesity). The patterns of multimorbidity were defined in this study, based on the three most prevalent clusters of chronic diseases.

### Analysis

Descriptive analyses were used to explain the variables in the study, including the prevalence of multimorbidity, the proportion of participants with each chronic disease, the average number of chronic diseases among individuals, and the distribution of individuals assigned to different numbers of chronic diseases. Prevalence-based analysis was used to rank the most prevalent chronic disease combinations (the common multimorbidity patterns). A chi-square test was used to examine the relationship between gender and/or age and the prevalence of these patterns. P for trend (p-trend), was used to test whether the variable showed a significance trend in response to change in another ordered variable. P-trend was used to measure two associations: one is between the prevalence of multiple chronic conditions and age (median age of participants with these diseases in different age groups); the other one is the distribution of number of chronic conditions and their prevalence. All statistical significance levels were defined as p-value less than 5%. Statistical analyses with drawn graphs were produced using STATA 16 with Excel 2016.

## Results

A total of 42 785 participants aged 23 to 96 were included in the study, with approximately 45.2% being men and 2.3% aged 60+ (3.7% of men were over 60) (see Table 1). About 10.3% of participants had two or more chronic diseases concurrently, indicating multimorbidity, notably more prevalent in males (around 14.8% vs 6.6% in females). Additionally, there was a positive linear association between age and multimorbidity prevalence (p-trend = 0.0111) in Table 1.

**Table 1.** Characteristics of the study population of multimorbidity in the Thai Cohort Study in 2013

	Number of people (n)	People with multimorbidity (n)	Proportion (%) (95% CI)
Male	19 330	2863	14.8 (14.3 to 15.3)
≤39 y	8251	530	6.4 (5.9 to 7.0)
40–59 y	10 371	2088	20.1 (19.4 to 20.9)
≥60 y	708	245	34.6 (31.1 to 38.2)
p-trend^a^	NA	NA	**0.0333**
Female	23 455	1538	6.6 (6.2 to 6.9)
≤39 y	13 687	383	2.8 (2.5 to 3.1)
40–59 y	9505	1073	11.2 (10.7 to 11.9)
≥60 y	263	82	31.2 (25.6 to 37.2)
p-trend^a^	NA	NA	**0.0106**
Total	42 785	4401	10.3 (10.0 to 10.6)
≤39 y	21 938	913	4.2 (3.9 to 4.4)
40–59 y	19 876	3161	15.9 (15.4 to 16.4)
≥60 y	971	327	33.7 (30.7 to 36.8)
p-trend^a^	NA	NA	**0.0111**

a p for trend <0.05.

**p-trend bolding**: The results of p for trend were statistical significance.

NA: not available; 95% CI: 95% confidential interval.

In Table 2, the average number of diseases per person rose from 0.53 for all ages to 1.20 for individuals over 60, exhibiting a linear increase with age (p-trend = 0.0120). The chronic conditions with high prevalence were metabolic syndrome-related conditions. Of these, obesity was most prevalent in both genders (total: 28.5%), followed by hyperlipidaemia (total: 13.2%) and hypertension (total: 7.2%) (see Table 2). Moreover, three common multimorbidity patterns were identified: hyperlipidaemia + obesity, hypertension + obesity, and hyperlipidaemia + hypertension + obesity.

**Table 2.** Number and proportion of participants with each chronic condition and the mean number of chronic diseases among individuals in the Thai Cohort Study in 2013

	Mean number of conditions													
	Diabetes (n (%))	High cholesterol/high blood lipids (n (%))	High bloodpressure (n (%))	Ischemic heart disease (n (%))	Cerebrovascular disease/stroke (n) (%)	Obesity (n) (%)	Liver cancer (n) (%)	Lung cancer (n) (%)	Stomach cancer (n) (%)	Colon-rectum cancer (n) (%)	Breast cancer (n) (%)	Kidney disease (n (%)	Total conditions (n)	Mean number of conditions (n ±SD)
Male														
All age	691 (3.6)	3188 (16.5)	2009 (10.4)	110 (0.6)	96 (0.5)	7089 (36.7)	18 (0.1)	12 (0.1)	38 (0.2)	24 (0.1)	8 (0.0)	177 (0.9)	13 460	0.70±0.02
≤39 y	75 (0.9)	619 (7.5)	309 (3.8)	11 (0.1)	13 (0.2)	2668 (32.3)	6 (0.1)	5 (0.1)	24 (0.3)	6 (0.1)	5 (0.1)	45 (0.6)	3786	0.46± 0.01
40–59 y	527 (5.1)	2354 (22.7)	1456 (14.0)	72 (0.7)	66 (0.6)	4166 (40.2)	10 (0.1)	7 (0.1)	14 (0.1)	14 (0.1)	3 (0.0)	113 (1.1)	8802	0.85±0.01
≥60 y	89 (12.6)	215 (30.4)	244 (34.5)	27 (3.8)	17 (2.4)	255 (36.0)	2 (0.3)	0	0	4 (0.6)	0	19 (2.7)	872	1.23±0.04
p-trend^a^	NS	NS	NS	NS	NS	NS	NS	NS	NS	NS	**0.0408**	NS	NA	**0.0441**
Female														
All age	350 (1.5)	2473 (10.5)	1059 (4.5)	25 (0.1)	65 (0.3)	5098 (21.7)	3 (0.0)	11 (0.1)	54 (0.2)	10 (0.0)	96 (0.4)	161 (0.6)	9405	0.40±0.02
≤39 y	87 (0.6)	712 (5.2)	211 (1.5)	7 (0.1)	19 (0.1)	2527 (18.5)	3 (0.0)	6 (0.0)	43 (0.3)	5 (0.0)	17 (0.1)	86 (0.6)	3723	0.27±0.01
40–59 y	244 (2.6)	1661 (17.5)	777 (8.2)	18 (0.2)	44 (0.5)	2472 (26.0)	0	5 (0.1)	11 (0.1)	4 (0.0)	78 (0.8)	72 (0.8)	5386	0.57±0.01
≥60 y	19 (7.2)	100 (38.0)	71 (27.0)	0	2 (0.8)	99 (37.6)	0	0	0	1 (0.4)	1 (0.4)	3 (1.1)	296	1.13±0.05
p-trend^a^	NS	**0.0135**	NS	NS	NS	**0.0006**	NS	NS	NS	NS	NS	NS	NA	**0.0316**
Total														
All age	1041 (2.4)	5661 (13.2)	3068 (7.2)	135 (0.3)	161 (0.4)	12 187 (28.5)	21 (0.1)	23 (0.1)	92 (0.2)	34 (0.1)	104 (0.2)	338 (0.8)	22 865	0.53±0.01
≤39 y	162 (0.7)	1331 (6.1)	520 (2.4)	18 (0.1)	32 (0.1)	5195 (23.7)	9 (0.0)	11 (0.1)	67 (0.3)	11 (0.1)	22 (0.1)	131 (0.6)	7509	0.34±0.01
40–59 y	771 (3.9)	4015 (20.2)	2233 (11.2)	90 (0.5)	110 (0.6)	6638 (33.4)	10 (0.1)	12 (0.1)	25 (0.1)	18 (0.1)	81 (0.4)	185 (0.9)	14 188	0.71±0.01
≥60 y	108 (11.1)	315 (32.4)	315 (32.4)	27 (2.8)	19 (2.0)	354 (36.5)	2 (0.2)	0	0	5 (0.5)	1 (0.1)	22 (2.3)	1168	1.20±0.04
p-trend^a^	NS	NS	NS	NS	NS	NS	NS	NS	NS	NS	NS	NS	NA	**0.0120**

a p for trend <0.05.

**p-trend bolding**: The results of p for trend were statistical significance.

NA: not available; SD: standard deviation; NS: not significant.

Table 3 indicated that individuals with four or more diseases were less common compared to those with one or two diseases (p-trend = 0.0330). This difference was not significant among those under 39 y but showed significance in the 40–59 y group (p-trend = 0.0168) and in the 60+ y group (p-trend = 0.0026). Among all participants, around 61.0% had no diseases, 28.7% had one condition, and around 7.2% had two diseases. Nonetheless, disease prevalence increased with age, with only about one-third of individuals aged 60 and above being disease-free.

**Table 3.** Distribution of number of chronic conditions in the Thai Cohort Study in 2013

		Age groups			
	All age, proportion (%) (95% CI)	≤39 y, proportion (%) (95% CI)	40–59 y, proportion (%) (95% CI)	≥60 y, proportion (%) (95% CI)	p-trend^a^
Male					
Number of conditions					
No condition	51.8 (51.1 to 52.5)	62.7 (61.6 to 63.7)	44.3 (43.3 to 45.2)	35.3 (31.8 to 38.9)	NS
One condition	33.9 (33.2 to 34.5)	30.9 (29.9 to 31.9)	35.6 (34.7 to 36.5)	30.1 (26.7 to 33.6)	NS
Two conditions	10.0 (9.6 to 10.4)	4.8 (4.4 to 5.3)	13.4 (12.8 to 14.1)	18.6 (15.8 to 21.7)	NS
Three conditions	3.8 (3.6 to 4.1)	1.4 (1.1 to 1.6)	5.3 (4.9 to 5.8)	10.5 (8.3 to 12.9)	**0.0466**
Four conditions	0.9 (0.8 to 1.1)	0.2 (0.1 to 0.3)	1.2 (1.0 to 1.5)	4.5 (3.1 to 6.3)	NS
More than four conditions	0.0 (0.0 to 0.2)	0.0 (0.0 to 0.1)	0.0 (0.0 to 0.3)	0.4 (0.4 to 2.0)	NS
p-trend^a^	**0.0182**	**0.0395**	**0.0115**	**0.0021**	NA
Female					
Number of conditions					
No condition	68.6 (68.0 to 69.2)	76.2 (75.5 to 76.9)	58.8 (57.8 to 59.8)	29.7 (24.2 to 35.6)	**0.0139**
One condition	24.8 (24.3 to 25.4)	21.0 (20.3 to 21.7)	29.9 (29.0 to 30.8)	39.7 (33.2 to 45.4)	NS
Two conditions	4.9 (4.6 to 5.2)	2.3 (2.1 to 2.6)	8.1 (7.5 to 8.7)	21.3 (16.5 to 26.7)	NS
Three conditions	1.3 (1.1 to 1.4)	0.4 (0.3 to 0.5)	2.4 (2.1 to 2.7)	8.8 (5.6 to 12.8)	NS
Four conditions	0.4 (0.3 to 0.5)	0.1 (0.1 to 0.2)	0.8 (0.7 to 1.0)	1.14 (0.2 to 3.3)	NS
More than four conditions	0.0 (0.0 to 0.1)	0.0 (0.0 to 0.1)	0.0 (0.0 to 0.1)	0	NS
p-trend^a^	0.0512	.0.0739	**0.0294**	**0.0062**	NA
Total					
Number of conditions					
No condition	61.0 (60.6 to 61.5)	71.1 (70.5 to 71.7)	51.2 (50.5 to 51.9)	33.7 (36.2 to 42.4)	NS
One condition	28.7 (28.3 to 29.1)	24.7 (24.2 to 25.3)	32.9 (32.2 to 33.5)	32.5 (29.6 to 35.6)	NS
Two conditions	7.16 (6.9 to 7.4)	3.3 (3.0 to 3.5)	10.9 (10.4 to 11.3)	19.3 (16.8 to 21.9)	**0.0182**
Three conditions	2.4 (2.3 to 2.6)	0.7 (0.6 to 0.9)	3.9 (3.6 to 4.2)	10.0 (8.1 to 12.1)	NS
Four conditions	0.6 (0.6 to 0.7)	0.1 (0.1 to 0.2)	1.0 (0.9 to 1.2)	3.6 (2.5 to 5.0)	**0.0320**
More than four conditions	0.1 (0.1 to 0.1)	0.0 (0.0 to 0.1)	0.1 (0.1 to 0.12)	0.7 (0.3 to 1.5)	NS
p-trend^a^	**0.0330**	0.0575	**0.0168**	**0.0026**	NA

a p for trend <0.05.

**p-trend bolding**: The results of p for trend were statistical significance.

NA: not available; 95% CI: 95% confidential interval; NS: not significant.

In Table 4, the three most common patterns of multimorbidity (based on the prevalence of these chronic conditions combination) were hyperlipidaemia + obesity, hypertension + obesity, and hyperlipidaemia + hypertension + obesity. The top was hyperlipidaemia + obesity, affecting 30.4% of individuals with multimorbidity (men: 29.8%, women: 31.5%). Following this, hypertension + obesity was the second most prevalent, accounting for 16.5% overall, with 15.9% of men and 17.5% of women experiencing multimorbidity. Hyperlipidaemia + hypertension + obesity was reported in 16.0% of men, 10.6% of women, totalling 14.1% in the multimorbid population (see Table 4).

**Table 4.** Prevalence of the three most frequent multimorbidity patterns (hyperlipidemia + obesity, hypertension + obesity, and hyperlipidemia + hypertension + obesity) among individuals in the Thai Cohort Study in 2013

Age groups	Number of patients (n)	Proportion among all participants (%) (95% CI)	Proportion (%) of patients among multimorbid population (95% CI)
**Hyperlipidemia + obesity**			
Male			
All ages	854	4.4 (4.1 to 4.7)	29.8 (28.2 to 31.5)
≤39 y	213	2.6 (2.3 to 3.0)	40.2 (36.0 to 44.5)
40–59 y	619	6.0 (5.5 to 6.4)	29.7 (27.7 to 31.7)
≥60 y	22	3.1 (2.0 to 4.7)	9.0 (5.7 to 13.3)
p-trend^a^	NA	NS	**0.0396**
Female			
All ages	484	2.1 (1.9 to 2.2)	31.5 (29.2 to 33.9)
≤39 y	140	1.0 (0.9 to 1.2)	36.6 (31.7 to 41.6)
40–59 y	328	3.5 (3.1 to 3.8)	30.6 (27.8 to 33.4)
≥60 y	16	6.1 (3.5 to 9.7)	19.5 (11.6 to 29.7)
p-trend^a^	NA	NS	**0.0301**
Total			
All ages	1338	3.1 (3.0 to 3.3)	30.4 (29.1 to 31.8)
≤39 y	353	1.6 (1.5 to 1.8)	38.7 (35.5 to 41.9)
40–59 y	947	4.8 (4.5 to 5.1)	30.0 (28.4 to 31.6)
≥60 y	38	3.9 (2.8 to 5.3)	11.6 (8.4 to 15.6)
p-trend^a^	NA	NS	**0.0411**
**Hypertension + obesity**			
Male			
All ages	455	2.4 (2.1 to 2.6)	15.9 (14.6 to 17.3)
≤39 y	102	1.2 (1.0 to 1.5)	19.3 (16.0 to 22.9)
40–59 y	315	3.0 (2.7 to 3.4)	15.1 (13.6 to 16.7)
≥60 y	38	5.4 (3.8 to 7.3)	15.5 (11.2 to 20.7)
p-trend^a^	NA	**0.0313**	NS
Female			
All ages	269	1.2 (1.0 to 1.3)	17.5 (15.6 to 19.5)
≤39 y	73	0.5 (0.4 to 0.7)	19.1 (15.3 to 23.4)
40–59 y	183	1.9 (1.7 to 2.2)	17.1 (14.9 to 19.4)
≥60 y	13	4.9 (2.7 to 8.3)	15.9 (8.7 to 25.6)
p-trend^a^	NA	NS	NS
Total			
All ages	724	1.7 (1.6 to 1.8)	16.5 (15.4 to 17.6)
≤39 y	175	0.8 (0.7 to 0.9)	19.2 (16.7 to 21.9)
40–59 y	498	2.5 (2.3 to 2.7)	15.8 (14.5 to 17.1)
≥60 y	51	5.3 (3.9 to 6.9)	15.6 (11.8 to 20.0)
p-trend^a^	NA	**0.0214**	NS
**Hyperlipidemia + hypertension + obesity**			
Male			
All ages	457	2.4 (2.2 to 2.6)	16.0 (14.6 to 17.4)
≤39 y	72	0.9 (0.7 to 1.1)	13.6 (10.8 to 16.8)
40–59 y	348	3.4 (3.0 to 3.7)	16.7 (15.1 to 18.3)
≥60 y	37	5.2 (3.7 to 7.1)	15.1 (10.9 to 20.2)
p-trend^a^	NA	NS	NS
Female			
All ages	163	0.7 (0.6 to 0.8)	10.6 (9.1 to 12.2)
≤39 y	19	0.1 (0.1 to 0.2)	5.0 (3.0 to 7.6)
40–59 y	128	1.4 (1.1 to 1.6)	11.9 (10.1 to 14.0)
≥60 y	16	6.1 (3.5 to 9.7)	19.5 (11.6 to 29.7)
p-trend^a^	NA	NS	NS
Total			
All ages	620	1.5 (1.3 to 1.6)	14.1 (13.1 to 15.2)
≤39 y	91	0.4 (0.3 to 0.5)	10.0 (8.1 to 12.1)
40–59 y	476	2.4 (2.2 to 2.6)	15.1 (13.8 to 16.4)
≥60 y	53	5.5 (4.1 to 7.1)	16.2 (12.4 to 20.7)
p-trend^a^	NA	**0.0158**	NS

a p for trend <0.05.

**p-trend bolding**: The results of p for trend were statistical significance.

NA: not available; 95% CI: 95% confidential interval; NS: not significant.

Among the three multimorbidity patterns (see Table 4), hyperlipidaemia + obesity was most prevalent in both male and female multimorbid patients under 39 y, constituting 40.2% and 36.6% (total: 38.7%), respectively. This pattern slightly decreased with age but lacked significant decline. Hypertension + obesity was less common in the over-60 age group, making up 15.6% of the multimorbid population. Hyperlipidaemia + hypertension + obesity prevailed in middle age and older multimorbid individuals, with the highest occurrence observed among males aged 40–59 y (16.7%) and females over 60 y (19.5%).

In the over 60 age group, hypertension + obesity prevalence reached 5.4% in men and 4.9% in women, notably higher in men. Meanwhile, hyperlipidaemia + hypertension + obesity in this age group was 0.9% higher in females compared to males in Table 4. Additionally, among individuals over 60, women (6.1%) were more likely than men (3.1%) to experience hyperlipidaemia + obesity. The prevalence of hypertension + obesity increased with age for all participants, demonstrating a significant upward trend (p-trend = 0.0214). Overall, men appeared to have a higher likelihood of experiencing all three multimorbidity patterns compared to women across all participants (see Table 4).

## Discussion

About one-third of participants over the age of 60 had two or more chronic conditions at the same time and nearly 70% were classified as having at least one disease in this study. Even amongst participants aged between 40 and 59 y multimorbidity prevalence was near 16.0%. About half of the population had at least one chronic condition, and one in seven suffered from two or more diseases. This suggests that multimorbidity may no longer be the ‘preserve’ of the elderly. This was also confirmed in studies in Canada and Northern Ireland that found an increasing number of younger and middle-aged people (from 25 to 60 y) were suffering from multimorbidity.^12,13^ However, such a phenomenon has not yet been shown in low- and middle-income countries, which may possibly be related to the lack of research on multimorbidity in these countries.^14^

In our study, young people in Thailand were more likely to suffer from multimorbidity pattern in terms of metabolic syndromes such as obesity and hyperlipidaemia, which may be related to high-oil, high-salt, and high-sugar diets, as well as sedentary lifestyle habits.^15^ However, the mechanism of multimorbidity in an increasing number of young and middle-aged people still needs to be elucidated by more research, and there is no clear understanding at this present time.^1,2^ Nevertheless, age is still the most important factor in multimorbidity, and the older people are, the more likely they are to develop it.^16^ Thus, increased susceptibility to multiple chronic conditions is also a characteristic of the ageing society.

The prevalence of multimorbidity was higher in men than in women in all age groups in this paper, contrary to the results of some other papers. In studies from Aragon and Catalonia^17^ and Bangladesh,^18^ the prevalence of multimorbidity was higher in females than in males in both older and relatively younger age groups. It has been suggested that physical multimorbidity may be more common in men and physical–mental multimorbidity more common in women, so that the different chronic conditions included in multimorbidity may also have some influence on gender-related outcomes.^19^

Psychiatric disorders were not included in our research, which may have had some impact on our gender-related results, which differed from other studies.^17,18,20^ However, some studies that did include psychiatric disorders, still found no significant difference in the prevalence of multimorbidity between men and women.^21^ It is also necessary to mention that in the TCS, the study population were more educated than the general population. In particular, the higher educational attainment of the women in the TCS may have caused some differences with Thai women as a whole.^3,9^ Thus, we could not determine which of these factors should play a dominant role.^19–21^ Gender may have an impact on multimorbidity and its patterns, but there are fewer articles addressing these aspects, particularly gender-related patterns of multimorbidity.^17^

The vast majority of patients with multimorbidity had two to three diseases in our study, which is similar to a study in Indonesia.^22^ Furthermore, the findings from studies in Indonesia,^22^ Thailand and the Philippines^23^ showed that metabolic syndromes including obesity, hypertension and hyperlipidaemia were among the most prevalent chronic conditions. And several of the most common combinations of chronic diseases in these studies were also related to obesity, hypertension and hyperlipidaemia.^22,23^ However, it is worth noting that most prevalent disease in our study was obesity, with one in three to four people being obese, which was similar to the national prevalence of obesity (BMI over 25 kg/m^2^) in Thailand.^24–26^

Prevalence increased with age for all three multimorbidity patterns in this research, which is also consistent with previous studies in Asia.^21–23^ One of the possible reasons is that obesity, hyperlipidaemia and hypertension are also chronic diseases whose prevalence would increase progressively with age.^27^ In addition, in the Yi et al. study,^28^ the prevalence of metabolic syndrome (including obesity, hyperlipidaemia and hypertension) was higher in men compared to all-age female participants. Nevertheless, the prevalence was higher in older women (over 60 y) than in men, whereas in the under 60 age group, the prevalence was higher in men than in women, which was also similar to our findings. The results in our study showed that the prevalence of the three most common multimorbidity patterns was higher in males than females, while in the patterns of multimorbidity of hyperlipidaemia + hypertension + obesity and hyperlipidaemia + obesity, the prevalence was higher in women than in men in the 60+ y age group.

## Strengths and limitations

This article was the first attempt to use age and gender stratification to explore the prevalence of multimorbidity and the patterns of multimorbidity that were common in the TCS. Although the method of data collection for diseases in this study was self-report, potentially producing recall bias, the research by Papier et al.^11^ revealed a high degree of concordance between the self-reported collected disease data in the TCS and the clinical assessments, which further supported the reliability and accuracy of our self-reported data.^9,11^ In this study, obesity was included as a chronic condition because obesity is considered an entry point for multimorbidity, and the inclusion of obesity as a pathological condition rather than a risk factor in the study of multimorbidity could provide an evidence base for the prevention and treatment of obesity-related multimorbidity.^22,29,30^

However, this article also had some shortcomings. First, this paper was a cross-sectional study, so it would be not possible to determine the causal relationship between age, gender and multimorbidity.^29,30^ The data of the study was collected in 2013, about a decade ago, thus multimorbidity patterns may change over the years. In addition, the other limitation was that there was a lack of information on mental illness as well as on the severity of chronic conditions in the TCS.^19^ Since the members in the study were open university students, their age distribution was generally younger and they were better educated than the Thai population in general.^9,10^

## Future research

It is imperative to take into account gender and age differences in future studies of multimorbidity and its patterns, especially those in which multimorbidity is associated with the metabolic syndrome. Conducting longitudinal studies is also essential to explore the causal relationship between gender, age and multimorbidity, along with different patterns of multimorbidity in Thailand.^1,2^ These studies can provide local governments with detailed information that can help them to develop more effective multimorbidity prevention and treatment programmes for people of different genders and ages, to achieve better preventive and therapeutic outcomes.^1,2^

### Conclusion

Metabolic syndrome-related factors were the most prevalent chronic conditions among multimorbidity. In addition, there were significant age and gender differences in the prevalence of multimorbidity and the most common of patterns of multimorbidity. People aged over 60 y were at highest risk for multimorbidity, while younger individuals under the age of 40 y tended to have the multimorbidity pattern of obesity and hyperlipidaemia. Moreover, in comparison to women, men showed a higher likelihood of multimorbidity, and multimorbidity patterns associated with metabolic syndrome (including obesity, hypertension and hyperlipidaemia) were also more prevalent among males. Therefore, it is necessary to consider gender and age differences in future studies of multimorbidity and its patterns, especially those addressing metabolic syndromes in Thailand. Research on multimorbidity should be stratified by sex and age to ensure a more comprehensive understanding of the situation in different population groups.

## Data Availability

Data may be requested from the authors if the reason is relevant and sufficient.
